# New Jersey State Dental Society

**Published:** 1891-06

**Authors:** 


### NEW JERSEY STATE DENTAL SOCIETY.—TWENTIETH ANNUAL SESSION.

(Continued from page 255.)

### DISCUSSION ON DR. R. C. NEWTON'S PAPER.

(For Dr. Newton's paper, see page 353.)

*Dr. B. F. Luckey.*—Mr. President and Gentlemen,—When I read on the programme that I was to open the discussion on this paper I was astonished, not having been forewarned and not having had an opportunity to read the paper since.

I do not think it would have made any difference if I had had opportunity to do so, for I do not see, upon listening to it, that there is anything in it that is open to discussion to any extent. As a dental paper from a medical source it is one worthy of commendation. As a general thing, such papers coming from medical sources are very apt to be loaded and surcharged with ideas that are fallacious. As a rule, medical men give so little attention and thought to dental subjects that when they do consider them they do so a good deal in the manner of the laity. The doctor has dealt to-day with the subject of "The Physiology and Hygiene of the Second Dentition," and it is a compilation of well-known facts that have been gathered apparently from reliable sources. But, so far as I remember, none of his remarks bring out anything entirely new, and he reduces the work of a dentist down to the use of a lancet. Almost all of the troubles he mentioned occur through retarded dentition, or irritation from retarded dentition. I think, perhaps, I can do nothing more interesting than to cite a case which has just recently passed through my hands to illustrate that if physicians were a little more conscientious in consulting with their brotherhood, the dental profession, they would not only confer a boon upon their patients, but also relieve themselves of a good deal of the odium which attaches to the mistakes which all professional men are liable to make.

A patient, probably thirty years of age, presented a very badly-swollen jaw. The swelling was of such a nature that the patient was unable to open his jaws sufficiently to permit of a proper examination of the whole of the mouth. He had been under the treatment of a physician for nearly three weeks, and was nearly exhausted from pain, suffering, etc. His physician told him there was dead bone back of it, that there was necrosis, and he would have to operate to relieve him. He came to see me about it. I could not with the little chance I had for exploration determine satisfactorily to my own mind what the trouble was, but I came almost positively to the conclusion that it was due to a wisdom tooth coming through in such a direction that it was not possible for it to get into position: it was tilted over in the ramus of the jaw and came directly against the distal surface of the second molar tooth. I informed the gentleman that, as far as I could judge, that was his trouble, that if he would open this abscess and allow it to discharge, and come and see me as soon as he could open his mouth properly, I might be able to help him. The abscess was opened and discharged freely, but the hard swelling did not subside, and the man was not able to open his mouth for two or three days. He suffered a great deal, and I advised him again and urged him not to poultice from the outside, his physician constantly recommending that process. I explained to him it would not relieve him, but the probability was he would have a scar on the outside of his face as long as he lived. I informed him after the second visit that I was sure there was no necrosis, and our only hope was to extract the second molar, which was perfectly sound, and allow the wisdom tooth to come in in that way. He did not feel willing to agree to that, and of course we could not perform any operation until his jaws could be opened. He came to me the second time some weeks after and I gave him the same advice, but he did not feel inclined to accept it; he said he would come when he could open his mouth; in the mean time he went back to his physician and made an appointment; they anæsthetized him, opened his face, went for the dead bone, and did not find it. The man recovered after awhile sufficiently to open his mouth and came to see me again; by that time the wisdom tooth had begun to show itself slightly, and I was satisfied thoroughly then of the diagnosis, and again advised him to submit to the operation; he did so and recovered entirely; but he has two horrible scars on the outside of his face which he will carry to his grave. There was a case of suffering for some two months or more, which could have been cured at the start, in all probability, had there been a consultation between the physician and the dentist.

As to the hygienic portion of the paper, I do not see that I can add anything to what the doctor has said as to the care and nutrition of the patient, excepting to advocate the constant and thorough use of the teeth and their cleansing. The teeth, like all other organs of the body, require use to get them in their proper condition. The selection of soft food and the avoidance of the use of the teeth creates a tendency, as similar causes do in all other organs, to degeneration, and although abrasion may be somewhat wearing on them, it still has a tendency to the hardening of the teeth.

*Professor C. N. Peirce.*—I speak because I wish to congratulate the New Jersey Society on having a physician read a paper before it, and I want to congratulate the author of the paper on the arrangement of his subject and his treatment of it, because I believe it is a very great step in advance of a large class of medical practitioners. The reading of the paper has given me a good deal of pleasure, and although perhaps he has not illustrated anything but what many of our leading dentists have been familiar with for years, yet it shows that the medical profession are recognizing the influence of the teeth in certain directions. It also adds greatly to the status of the dental profession in the eyes of the medical profession when it is recognized that the teeth themselves are capable of producing such systemic disturbances. I could make no criticism at all of the paper excepting as to a remark that the character of the first molars and the influence of dentition was quite as great in the development of a child through youth and to manhood or womanhood as hereditary influences are. While I recognize the influence of dentition very fully, yet I want to say that hereditary conditions are in advance of the results accomplished by individuals. There may be temporary influences during the period of dentition that will effect the development of the teeth in an infant, and will necessarily cause great disturbances in the eruption of the permanent teeth. As the doctor is well aware, when a child is born it has the twenty deciduous teeth largely calcified; those teeth are influenced by the action of the health of the mother during the period of gestation. During the first year of life we have the incisors and first molars undergoing calcification. The first molars at birth often have the point of the cusps already completed. The health of the mother must necessarily produce imperfect development in those first molars if it is bad, and unless that child has peculiar influences about it during the first two years of its life all the permanent teeth will be imperfectly calcified and oftentimes produce great disturbance in their eruption in consequence of the imperfect nourishment of the infant.

Otherwise, I have to say amen to every word of the paper, with just that little criticism.

*Dr. J. A. Osmun.*—I have nothing to add excepting words of commendation for the paper, and as I am delighted with it I can only re-echo the sentiment of Dr. Peirce,—that dentists and physicians are becoming brothers in a common cause, and the time is coming, I hope, when they will stand shoulder to shoulder in their efforts to relieve the sufferings of their patients. It should be so.

The doctor's paper points out in a broad way the influences that come from first and second dentition. From a physician's standpoint he deals with the body as an entirety, but he also feels that he should have the benefit of consultation with his brethren who make a study of this particular portion of the body. I say it is a happy omen of the time when we shall stand shoulder to shoulder in our efforts to alleviate pain.

*Professor Charles Mayr.*—Professor Peirce's remarks have suggested to me a most interesting subject, namely, the question of how the various cells in the child during the period of gestation are connected with the corresponding cells in the mother. You know there are two theories, one is that from every tooth in the mother's mouth there goes a nerve-filament to the growing tooth of the child, and I think the observation is not uncommon that women about that time feel various organs getting out of order; first the stomach is wrong, and this is wrong and the other is wrong, and then the teeth are wrong. I think that is the first shock of the connection, and afterwards it is normal. We can remember our experiences when we were first thrown in the water to learn to swim; we remember it all our life long, but afterwards we tumble in a dozen times and forget all about our sensations, so that I think the moment a woman in that condition feels her teeth aching the connection is made and the teeth begin to calcify, and the child too has to get its starting-point from the lime salts of its mother's teeth.

I believe myself too much in the individuality of atoms; in my opinion there are no two atoms exactly alike, every atom has its individuality.

The other theory is that no such connection exists, but an impulse of wave motion proceeds from the teeth of the mother towards the child. Which of the two theories is correct is hard to tell.

*Dr. Sanger.*—Suppose the mother's teeth are false, what then?

*Professor Mayr.*—Well, of course the nerves of the teeth are still there, the teeth and the endings and all that; the nerve-endings which formed the teeth before are still there unless the whole tooth and nerve is destroyed.

*Dr. Walker.*—Does Professor Mayr think that the lime-salts are gradually absorbed from the bony system of the mother and pass through the circulation to the child?

*Professor Mayr.*—As I said, there are two theories, one being that only the wave motion is transmitted, and the other that entire particles from the part are transmitted down to the corresponding part in the child.

*Professor Peirce.*—There are a great many mothers who bear children without any detriment to the teeth. The mouth is kept in a perfectly healthy condition all through the period of gestation, and not only one, but sometimes ten or twelve children are borne, and the teeth are always in good condition. On the other hand, we find mothers who become exhausted and are not able to produce sufficient nutrition to build up the embryo, and so the deficiency arises through the inefficiency of the mother.

I know of an instance where, in the case of an infant, the original teeth were entirely deficient of enamel. The permanent teeth came through in no better condition, and the difficulty was that the mother had just that trouble during the period of gestation. The child never had good teeth because, during the period of gestation, there was no ability to build up those structures.

*Dr. R. C. Newton.*—Our indiscretions sometimes serve us well. I think I read that line concerning hereditary wrong, but it drew forth a very interesting talk from Professor Peirce. I think it all goes to show the main contention of the paper is right,—that an immense amount of good can be done if you treat those matters intelligently and at the right time. Nine times out of ten it seems to me the constitution is good enough if the people having the care of children knew what was needed. Doctors have not given enough attention to the teeth; they do not care enough about it. Until within a few years they were brought up to consider the body as a receptacle for drugs which should remedy existing evil. The whole tendency of modern thought is to prevent the evil rather than to go upon the idea that for all troubles there is a remedy. As Professor Mayr has said, when we are able to restore decay we shall have discovered the elixir of life. None of us believe we shall ever find the elixir of life, but a little elixir of common sense in the bringing up of children would be a very good thing.

*Dr. J. A. Osmun.*—That is the idea I tried to bring out yesterday. The public ought to be better educated, and the province of dental and medical societies is to disseminate more knowledge among the laity.

*Dr. Newton.*—That is the view I have always taken, and for that reason I have always been willing to do all I could in that direction.

On motion, the paper of Dr. Newton was passed.

Adjourned until 8 P.M.

(To be continued.)

